# Do COVID-19 Conspiracy Theory Beliefs Form a Monological Belief System?

**DOI:** 10.1017/S0008423920000517

**Published:** 2020-05-21

**Authors:** Joanne M. Miller

**Affiliations:** Political Science and International Relations, University of Delaware, 347 Smith Hall, 18 Amstel Ave., Newark, Delaware 19716, USA

## Abstract

Along with criticisms of the U.S. government's response to the COVID-19 pandemic, the disruptions to home, work, and school life resulting from social distancing orders recommended by public health experts, as well as the uncertainty about how long the disruptions will be necessary and when (if ever) we will have a vaccine, have come COVID-19 conspiracy theories (CTs).

## Introduction

Along with criticisms of the U.S. government's response to the COVID-19 pandemic, the disruptions to home, work, and school life resulting from social distancing orders recommended by public health experts, as well as the uncertainty about how long the disruptions will be necessary and when (if ever) we will have a vaccine, have come COVID-19 conspiracy theories (CTs).

CTs are beliefs that powerful actors are engaging in wide-ranging secret (often illegal or ambiguously legal) activities for their own personal gain (Miller et al., 2016). Uscinski et al. (2020) find that 29 per cent of respondents in a recent, nationally representative sample agree that the threat of COVID-19 has been exaggerated to damage President Trump and 31 per cent agree that the virus was purposefully created and spread. A recent YouGov/The Economist poll found that 13 per cent believe it is a hoax. Such beliefs can have negative health effects; for example, Motta et al.'s (2020) data suggest that individuals who endorse conspiracy theories about COVID-19 are less likely to accept public health experts’ warnings about the severity of the crisis.

The motives that give rise to CT beliefs—conspiratorial thinking (the tendency to view events as the product of a conspiracy; Uscinski and Parent, 2014), denialism (the tendency to deny official/authoritative accounts of events; Uscinski et al., 2020), partisan-motivated reasoning (the desire to bolster/protect one's worldview; Miller et al., 2016; Pennycook et al., 2020), and uncertainty/powerlessness (van Prooijen and Acker, 2015)—are all certainly activated during the current pandemic. As such, it is not surprising that specific COVID-19 CTs are predicted by these motives (Uscinski et al., 2020).

To gain a better understanding of how to counteract COVID-19 CTs, it is important not only to examine the predictors of individual CT beliefs, but also to explore their underlying structure. A robust finding in the study of CTs is that belief in one predicts belief in others (Goertzel, 1994). In essence, CTs form a “unitary, closed-off worldview in which beliefs come together in a mutually supportive network known as a monological belief system” (Wood et al., 2012: 767). When CTs fit a similar theme, this makes sense: belief in one acts as “evidence” for another, and vice versa. However, Wood et al. (2012) demonstrate that people are quite willing to believe contradictory CTs (for example, that Osama Bin Laden is still alive *and* that he was already dead when the U.S. raid took place), and also that a belief system of even incompatible CTs is “held together” by the higher-order belief that government officials are often deceptive.

To the extent that COVID-19 CTs—even contradictory ones—form a monological belief system, attempts at debunking them individually will likely fail. A more effective approach may be to tackle the higher-order needs/beliefs that give rise to the belief system in the first place. Higher order beliefs, such as conspiratorial thinking, denialism, and distrust of government, are certainly likely culprits. But in the context of a pandemic, uncertainty looms large. According to a Pew survey conducted March 19–24, 2020, 43 per cent of Americans reported feeling anxious most of the time or occasionally over the previous week. And as of May 16, 2020, the Civqs rolling cross-section survey indicates that 61 per cent of Americans are extremely or moderately worried about a coronavirus outbreak in their local area. Such situationally induced uncertainty may lead to the type of explanation-seeking that would cause people to accept multiple, even contradictory, CTs.

This study has two goals: (1) to assess whether COVID-19 CTs that have become part of the pandemic discourse form a monological belief system and (2) to examine whether personal uncertainty binds CT beliefs together.

## Material and Methods

U.S. adults (n = 3,019) recruited via Lucid Theorem completed an online survey between April 24 and 28, 2020. Lucid provides a quota sample (using U.S. Census benchmarks) of individuals who have agreed to participate in scientific research. Data were also weighted based on the 2018 Current Population Survey (CPS) benchmarks for education, income, sex, race, and ethnicity to more accurately represent the U.S. population (see Appendix A, available online in the Supplementary Materials, for a comparison of sample characteristics with the CPS).

The survey assessed belief in the CTs described in Table 1 that have been recently circulating (Lynas, 2020). The order of the CTs was randomized across respondents. Responses were coded from 1–4 (definitely not; probably not; probably; definitely); higher numbers equal greater belief.

**Table 1:** COVID-19 conspiracy theories

Variable label	Question wording
BioChina	The virus is a biological weapon intentionally released by China.
AccChina	The virus was accidentally released by China.
AccUS	The virus was accidentally released by the U.S.
Scientists	Scientists are exaggerating the seriousness to make President Trump look bad.
Media	The media are exaggerating the seriousness to make President Trump look bad.
Hoard	Democratic governors are hoarding ventilators to make President Trump look bad.
Tests	Democratic governors are not distributing coronavirus tests to make President Trump look bad.
5GTech	5 G technology is causing the coronavirus to spread faster.
NotReal	The coronavirus isn't real.
Gates	Former Microsoft CEO Bill Gates is creating a tracking device to be injected with the coronavirus vaccine.
WorldPop	The coronavirus was intentionally created to reduce the world's population.

To examine the psychological/political correlates of COVID-19 CT beliefs, the survey included measures of partisanship, conspiratorial thinking, and uncertainty. *Partisanship* was coded into two dummy variables, one for Republicans/leaners vs. everyone else and one for pure Independents vs. everyone else. Democrats/leaners are the comparison group. *Conspiratorial thinking* was measured via a four-item scale (Uscinski et al., 2016). *Personal uncertainty* was measured via a three-item scale that assessed how uncertain people felt about themselves, their place in the world, and their future. See Appendix B, available online, for complete question wordings. All independent and control variables were recoded to range from 0–1.

## Results and Discussion

### COVID-19 CT Beliefs Are Widespread

**Figure 1:**
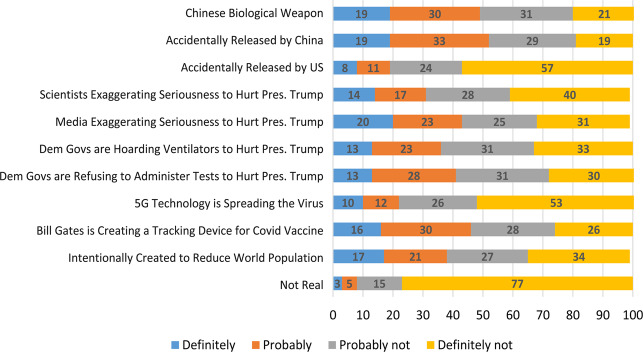
Distribution of COVID-19 CT beliefs.

**Figure 2:**
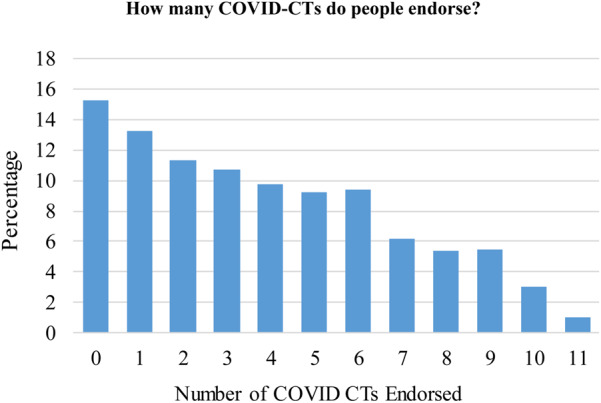
The unweighted percentages of people who endorse multiple CTs, combining the probably and definitely response options.

Figure 1 displays the distribution of responses to the 11 CT questions. Consistent with Uscinski et al. (2020), a striking percentage (between 3% and 19%) of respondents believe that each of these conspiracies is definitely occurring. Whereas only 8 per cent believe that the virus is probably (5%) or definitely (3%) not real, 49 per cent and 52 per cent report that they believe the virus was either probably or definitely intentionally created (as a biological weapon) or accidentally released by China. Fewer people believe it was accidentally released by the U.S. or that the virus is not real. Figure 2 graphs the total number of CTs respondents endorsed. Approximately 85 per cent believe more than one; 60 per cent believe three or more, and 30 per cent believe at least six.

### COVID-19 CT Beliefs Form a Monological Belief System

Not only are these beliefs widespread, but they are also interrelated. As Table 2 shows, the CTs are moderately to strongly positively correlated (all are statistically significant at p < .001). At the high end, the Scientists and Media CTs are correlated at .63. At the low end, the NotReal and AccChina CTs are correlated at .11. Given the floor effect in belief in the NotReal CT, it is not surprising that the correlations between it and the others are among the weakest.

**Table 2:** Correlations between the conspiracy theories

	BioChina	AccChina	AccUS	Scientists	Media	Hoard	Tests	5GTech	Gates	WorldPop	NotReal
BioChina	*										
AccChina	.43	*									
AccUS	.27	.26	*								
Scientists	.39	.27	.33	*							
Media	.37	.30	.25	.63	*						
Hoard	.46	.30	.34	.54	.58	*					
Tests	.34	.22	.22	.42	.41	.52	*				
5GTech	.38	.24	.47	.43	.33	.41	.24	*			
Gates	.40	.22	.31	.32	.30	.36	.25	.42	*		
WorldPop	.56	.29	.39	.41	.36	.46	.33	.48	.39	*	
NotReal	.14	.11	.27	.36	.28	.25	.26	.31	.18	.27	*

To formally test whether the 11 COVID-19 CT beliefs form a monological belief system, a principal factor analysis (PFA) was performed on the correlation matrix. The PFA yielded one factor with an Eigenvalue greater than 1 (3.96); the proportion of variance explained by the factor is .96 (the Eigenvalue for the second factor is .50). All 11 CTs positively load on this single factor, with factor loadings ranging from .40 to .73. Both the correlation matrix and the factor analysis provide strong support for the notion that the COVID-19 CTs are a monological belief system.

### Contradictory CTs Are Positively Related, Even When Controlling for Standard Predictors

On one hand, such a monological belief system could arise because the individual beliefs within it are mutually reinforcing. In other words, one CT belief becomes “evidence” for others (Goertzel, 1994). However, even the most obviously contradictory conspiracy theories are positively correlated, such as BioChina and AccUS (.27), AccChina and AccUS (.26), and BioChina and AccChina (.43). Fifteen per cent of respondents simultaneously believe that the BioChina and AccUS CTs are probably/definitely true; 15 per cent believe that both the AccChina and AccUS CTs are probably/definitely true, and 34 per cent believe that both the BioChina and AccChina CTs are probably/definitely true.

**Figure 3:**
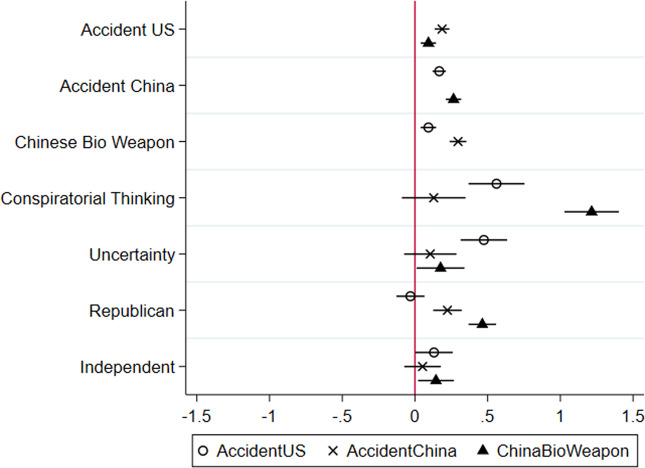
The unstandardized regression coefficients from ordinary least squares models that include controls for age, education, income, gender, race, and ethnicity. The dots represent the coefficients and the lines represent the 95 per cent confidence intervals. See Appendix C, available online, for the full models.

These results hold in multivariate models controlling for standard predictors (see Figure 3; NotReal was not included in these models due to lack of variance; see Figure 1); mutually incompatible CTs are statistically significant, positive predictors of one another. Specifically, endorsement of the theories that the virus was accidentally released by China, the virus was accidentally released by the U.S., and that it is a biological weapon intentionally released by China are statistically significant, positive predictors of one another. People are quite willing and able to endorse contradictory COVID-19 CTs.

Consistent with past research, conspiratorial thinking and uncertainty are statistically significant, positive predictors of all three CTs. Republicans are more likely than Democrats to believe that the virus was accidentally released by China and that it is a Chinese biological weapon, and Independents are more likely to believe all three CTs than Democrats.

### Personal Uncertainty Moderates the Effect of a CT Belief on a Contradictory CT Belief

**Figure 4:**
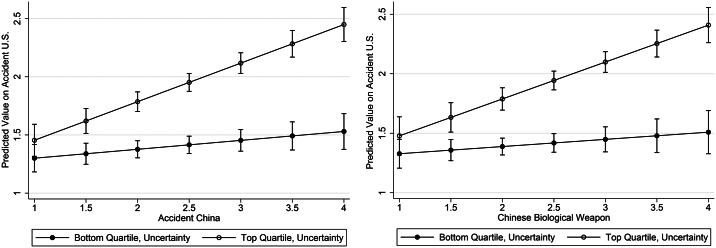
Personal uncertainty moderating the effect of AccChina and BioChina on AccUS. Predicted values for the left panel are derived from Model 1 in online Appendix C. The interaction and the effects of AccChina at the lowest and highest quartiles of uncertainty are all statistically significant (b = .45, p < .001; b = .08, p = .05; and b = .33, p < .001, respectively). Predicted values for the right panel are derived from Model 2 in online Appendix C. The interaction and the effect of BioChina at the highest quartile of uncertainty are statistically significant (b = .39, p < .001; b = .31, p < .001). The effect of BioChina at the lowest quartile of uncertainty is not significant (b = .06, n.s.).

To examine whether the COVID-19 belief system is driven by personal uncertainty, uncertainty was interacted with AccChina, BioChina, and AccUS predicting one another (in separate models). If the belief system is held to together by the situational induction of personal uncertainty surrounding the pandemic, then the relationship between mutually contradictory CTs should be stronger for those higher in uncertainty than their counterparts. To display the shape of the interactions, Figure 4 show the effects AccChina and BioChina for people in the bottom and top quartiles of uncertainty. Consistent with expectations, this is exactly the case (results for the AccChina and BioChina dependent variables are similar; see Appendix D, available online). In sum, the COVID-19 belief system is a more tightly knit structure (that is, it is more constrained) for those who are currently experiencing a great deal of uncertainty, likely due to the pandemic itself.

## Conclusion

Taken together, these findings provide strong support for the theory that COVID-19 CTs form a monological belief system. Not only are the CTs highly correlated, but a large majority believe more than one. Moreover, mutually contradictory CTs are positively related to one another, even when standard predictors of CT beliefs are taken into account. Finally, this belief system is more constrained for people who are currently experiencing a great deal of uncertainty. Two caveats to these conclusions are in order. First, a full accounting of the variety of COVID-19 CTs that are currently circulating was beyond the scope of this manuscript. It is possible that some of the ones out there that were not included in this study do not fit within this monological belief system. Second, it is possible that some of the CT responses represent expressive responding rather than true beliefs (Schaffner and Luks, 2018). As such, the distribution of responses to the CT questions may be biased upward. However, widespread expressive responding would likely attenuate relationships between CT beliefs and standard predictors evidenced here.

The breadth, and, for all intents and purposes, interchangeability of these beliefs may make attempts to debunk them by tackling each one individually akin to a futile game of whack-a-mole. Such attempts may be effective in the short term, but if the psychological needs that give rise to the beliefs in the first place remain intact, then a debunked CT will likely be replaced by another. A more effective, long-term strategy may be to address the political/psychological needs that give rise to the belief system in the first place. Alleviating situationally induced uncertainty, powerlessness, and concomitant anxiety (for example, van Prooijen, 2018) may be a promising antidote.

## Supplementary material

For supplementary material accompanying this paper visit http://dx.doi.org/10.1017/S0008423920000517. click here to view supplementary material
